# Disc Diffusion Testing of Azithromycin Against Clinical Isolates of Typhoidal Salmonellae: A Diagnostic Conundrum

**DOI:** 10.7759/cureus.16777

**Published:** 2021-07-31

**Authors:** Muhammad Shoaib, Luqman Satti, Ashfaq Hussain, Nazia Khursheed, Saba Sarwar, Abid H Shah

**Affiliations:** 1 Medical Microbiology, Pakistan Navy Ship (PNS) SHIFA Hospital, Bahria University Medical and Dental College, Karachi, PAK; 2 Departmetnt of Microbiology, The Indus Hospital, Karachi, PAK; 3 Preventive Medicine, Pakistan Navy Ship (PNS) SHIFA Hospital, Bahria University Medical and Dental College, Karachi, PAK

**Keywords:** azithromycin, covid-19, minimum inhibitory concentration, disc diffusion testing, extremely drug resistant typhoid

## Abstract

Introduction: Typhoid remains a major healthcare problem in low and middle-income countries. The emergence of extremely drug-resistant (XDR) typhoid strains from the Indian subcontinent has led to very limited therapeutic options. Azithromycin being the only oral option for XDR typhoid faces a threat of rapid resistance due to its overuse after the COVID-19 pandemic.

Objective: To evaluate the reliability of azithromycin disc diffusion testing against clinical isolates of typhoidal salmonellae in comparison with E-test minimum inhibitory concentrations (MICs).

Study design: This is a cross-sectional validation study.

Place and duration of the study: The Department of Microbiology, Pakistan Navy Ship Shifa hospital, Karachi from June 1 to December 31, 2020.

Methodology: Antimicrobial susceptibility was performed by Kirby Bauer disc diffusion method for 60 isolates including *Salmonella enterica* ser. Typhi and Paratyphi A using Clinical Laboratory Standard Institute (CLSI) guidelines. MICs by the E-test method were determined for Azithromycin only.

Results: A significant proportion of the isolates (55%) had high azithromycin MIC in the wild-type distribution range (8-16 µg/ml). Ten (16.6%) isolates showed false resistance, i.e., zone diameter <13 mm by disc diffusion method when compared to E-test MIC results. Isolates with MICs close to breakpoint, i.e., 16 µg/ml were more likely to show discordant results. The sensitivity, specificity, negative predictive value, positive predictive value, and diagnostic accuracy of the disc diffusion method versus E-test were 100%, 83%, 100%, 9%, and 83%, respectively.

Conclusions: Disc diffusion method as recommended by CLSI is not reliable for azithromycin susceptibility testing particularly for isolates with high MICs in the susceptible range. The E-test method may be a better alternative to disc diffusion provided appropriate training is done prior to its application.

## Introduction

Enteric fever is caused by typhoidal salmonellae, i.e., *Salmonella typhi* and *Salmonella paratyphi* A, B, and C [1]. *S. typhi* is exclusively a human pathogen. Typhoid fever is a cause of significant morbidity and mortality particularly in low and middle-income countries with poor quality of potable water and sanitation [2]. The disease is transmitted by contaminated food and water. It is a worldwide problem with endemic areas spread from South America, sub-Saharan Africa, the Middle East to South Asia and the Far East. The global incidence of the disease is estimated to be more than 11 million cases and 128,000 deaths annually [3].

Over the past four decades, the evolution of antimicrobial resistance in typhoidal salmonellae has rendered various classes of antibiotics ineffective leading to the emergence of multidrug-resistant (MDR) strains [4]. Fluoroquinolones resistance has been increasingly recognized owing to the emergence of a subclade of the H58 *S. typhi* (MDR) strain [5]. The acquisition of a plasmid-encoded extended-spectrum beta-lactamase (ESBL) gene blaCTX M-15 has further aggravated the situation by conferring resistance to ceftriaxone, leading to the emergence of extremely drug-resistant (XDR) strains [6]. XDR *S. typhi* is a continuous nuisance for the people of Pakistan and a global threat owing to ever-increasing international travelling. Currently, Pakistan is facing a dual ongoing epidemic of typhoid and COVID-19 [7].

Resistance to multiple classes of antibiotics has left clinicians with very limited options for the treatment of XDR Typhoid. Azithromycin is the only oral option available while meropenem being the only intravenous choice [8]. Azithromycin resistance in *S. typhi* isolates from India has been reported as early as 1999 and later in travelers from Asia [9,10]. Reports of Azithromycin resistance have emerged from several parts of the world especially typhoid endemic countries including India, Nepal, and Bangladesh among others [11-13]. Lack of antimicrobial stewardship practices in low and middle-income countries with over-the-counter availability of antimicrobials is a contributing factor to the increasing antimicrobial resistance [14]. Recently, owing to large-scale empirical use of azithromycin after the COVID-19 pandemic, there are concerns about its rising resistance and it is feared that we may soon run out of treatment options for XDR typhoid [15].

Moreover, antimicrobial susceptibility testing of azithromycin for typhoidal salmonellae has faced some challenges over time. According to Clinical Laboratory Standard Institute (CLSI) M100 standards 2020, azithromycin is still an investigational drug only for *S. enterica* ser. Typhi and the breakpoints are available based on minimum inhibitory concentration (MIC) distribution data and limited clinical data [16]. The earlier versions of the same document included epidemiological cut-off values for wild-type *S. typhi* isolates. Several studies have mentioned the discordance between disc diffusion and azithromycin MICs for typhoidal salmonellae [8,17,18].

Due to the scarcity of treatment options, it has become mandatory to adopt a reliable method for azithromycin susceptibility [19]. Therefore, this study was conducted to see the reliability of the disc diffusion method against the E-test method for clinical isolates of *S. typhi* in our setup.

## Materials and methods

This cross-sectional validation (diagnostic accuracy) study was conducted in the Department of Microbiology, PNS Shifa Hospital, Karachi from June 2020 to December 2020. Permission was obtained from Institutional Ethical Committee. *S. typhi* and *S. paratyphi* A isolates were collected from a tertiary care hospital by random consecutive sampling. Blood samples collected from febrile patients with suspected typhoid fever were inoculated into blood culture bottles containing brain heart infusion (BHI) enrichment media and incubated in an automated blood culture system at 37 °C. Bottles that flagged positively were subcultured on appropriate media including blood agar and MacConkey agar. The identification of the isolates was confirmed by colony morphology on differential media, biochemical reactions (API 20E, BioMérieux, Marcy-l'Étoile, France), and agglutination with type-specific antisera (MAST® ASSURE, UK).

Disc diffusion susceptibility

Antibiotic susceptibility testing was performed by Kirby Bauer method for antimicrobials using the Clinical Laboratories Standards Institute (CLSI) M100 standards, 2020 [16]. Overnight growth from blood agar was used to make 0.5 McFarland suspension for each isolate. The suspensions were inoculated onto 90 mm Mueller Hinton agar plates for disk diffusion susceptibility. The tests for disc diffusion inhibition zones were performed in duplicate for each isolate and the mean of the two values was used as the zone to interpret the result. American Type Culture Collection (ATCC) strains, i.e., *Escherichia coli* ATCC 25922, *Pseudomonas aeruginosa* ATCC 27853, and *Staphylococcus aureus* ATCC 25923 (for *S. enterica* ser. Typhi azithromycin disk diffusion testing only) were used for quality control as per CLSI standards. For azithromycin, isolates with zone diameter ≥13 mm were considered susceptible while those with zone diameter ≤12 were considered resistant by the disc diffusion method.

Azithromycin MICs

Minimum inhibitory concentrations were determined for azithromycin only by E-strip (BioMérieux) method with appropriate controls. To eliminate reader bias/error, MICs of all the isolates were recorded by using a second reader system. Bacterial suspensions were the same as used for disc diffusion susceptibility with turbidity equivalent to 0.5 McFarland for each isolate. The tests were duplicated and the MICs were recorded as the higher of the two values for each isolate. CLSI guidelines were used to interpret Azithromycin susceptibility, i.e., sensitive ≤16 µg/ml and resistant ≥32 µg/ml [16].

Errors in susceptibility

In this study, we recorded the errors in susceptibility as a very major error if false susceptible result by disc diffusion compared to the MIC value, and as a major error, if false resistant result produced by disc diffusion compared to MIC value and as minor errors if a difference of >2 mm in disc diffusion diameters on repeat testing.

Statistical analysis

The performance of disc diffusion was determined by calculating sensitivity, specificity, positive predictive value, negative predictive value, and accuracy. Sensitivity in our study represents the ability of a method to detect true resistant isolate to azithromycin while specificity means true sensitive isolate.

## Results

**Figure 1:**
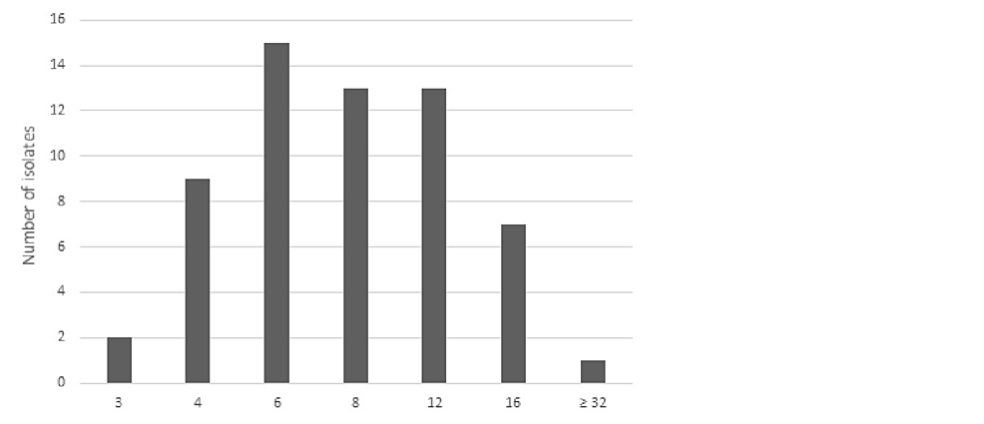
Azithromycin minimum inhibitory concentration distribution µg/ml (n=60)

A total of 60 isolates of Salmonella; *S. typhi* (n= 52) and *S. paratyphi* A (n=8) were tested. For azithromycin susceptibility, since CLSI guidelines 2020 describe the interpretive criteria only for *S. typhi*, we used the same criteria for *S. paratyphi* A. Among all the isolates 30 (50%) were XDR, 33 (55%) isolates had azithromycin MICs between 8 and 16 µg/ml (Figure 1). Only one XDR isolate had a very high MIC (96 µg/ml) with a disc zone of 9 mm and was reported as azithromycin resistant (true resistant). Ten isolates had discordance between disc diffusion and MIC susceptibility results with major errors, i.e., false resistance by disc diffusion method compared to MIC value. The isolates with MICs between 8 and 16 µg/ml were more likely to have discordant results. No discordance with disc diffusion results was seen for isolates having MICs between 3 and 6 µg/ml (Table 1). The sensitivity, specificity, negative predictive value, positive predictive value, and diagnostic accuracy of the disc diffusion method versus the E-test method are shown in Table 2. The sensitivity, specificity, negative predictive value, positive predictive value, and diagnostic accuracy were calculated using the following formulas while TP is true positive, TN is true negative, FP is false positive, and FN is false negative. (i) Specificity = (TN/TN+FP) × 100, (ii) sensitivity = (TP/TP+FN)× 100, (3) positive predictive value = (TP/TP+FP) × 100, (4) negative predicted value = (TN/TN+FN) × 100, (5) diagnostic accuracy = (TP+TN)/(TP+FP+FN+TN)×100.

**Table 1:** Comparison of the E-test and disc diffusion for discordant results (n=10) R: resistant, S: susceptible.

No.	Isolate	Disk diffusion susceptibility		MIC susceptibility	
		Zone (mm)	Interpretation	(µg/ml)	Interpretation
1	S. typhi	12	R	12	S
2	S. typhi	11	R	16	S
3	S. typhi	11	R	12	S
4	S. typhi	10	R	12	S
5	S. typhi	11	R	16	S
6	S. typhi	12	R	8	S
7	S. typhi	12	R	12	S
8	S. typhi	11	R	12	S
9	S. typhi	10	R	16	S
10	*S. paratyphi* A	9	R	16	S

**Table 2:** Comparison of results of disc diffusion method with E-test (n=60) NPV: negative predictive value, PPV: positive predictive value, DA: diagnostic accuracy.

Disc diffusion method	E-test		Performance of disc diffusion method				
	Resistant	Susceptible	Sensitivity	Specificity	NPV	PPV	DA
Resistant	1	10	100%	83%	100%	9.0%	83.6%
Susceptible	0	49					

## Discussion

A decade ago, azithromycin was not the treatment of choice for typhoid due to the availability of other options. There was a limited clinical experience with this drug for typhoid and susceptibility breakpoints were not defined. Since the emergence of XDR *S. typhi*, azithromycin has gained importance as the only oral option. After several reports of resistant isolates with MICs ≥32 µg/ml and clinical failure with azithromycin, it is feared that we may soon run out of this option. Several publications and editorials have raised concerns of increased azithromycin resistance due to its unjudicial use as a broad spectrum respiratory antimicrobial after the COVID-19 pandemic [15,20,21].

A very limited data from Pakistan are available on azithromycin MICs in typhoidal salmonellae. A study by Iqbal et al. included typhoidal salmonellae isolated from patients with suspected typhoid fever between September 2016 and September 2019 from two Hospitals in Karachi, Pakistan [18]. Among 2104 *S. typhi* isolates, one had high azithromycin MIC (12ug/ml) but was in the susceptible range. Another study by Klemm et al. included over 80 XDR *S. typhi* isolates mostly from an outbreak in Hyderabad, Pakistan that started in November 2016 [22]. In this study, only one isolate had MIC 8 µg/ml. There have been few reports of azithromycin-resistant isolates from Pakistan [23]. A recent study from Lahore reported one isolate with MICs 64 µg/ml [24]. In our study, we found a significant proportion (55%) of the isolates with MICs 8-16 µg/ml and one azithromycin resistant XDR isolate with MIC 96 µg/ml.

In another study, Khan et al. studied 100 isolates of typhoidal salmonellae collected from a tertiary care hospital in India between 2013 and 2017. In this study, mean azithromycin MIC had increased from 5 µg/ml in 2013 to 24 µg/ml in 2017. They reported discordance between azithromycin disc diffusion and E-test results and concluded that using disc diffusion guidelines by CLSI may result in misreporting of some isolates as resistant [17]. Similarly, Iqbal et al. also demonstrated discordance between azithromycin disc diffusion and E-test MICs results for five isolates [18]. In our study, we observed discordance in ten isolates; false resistance by disc diffusion method. We also found that discordance between disc diffusion and MIC results was more likely to be observed in the isolates with MICs at or near the breakpoint, i.e., 8-16 µg/ml. The findings of our study were consistent with Iqbal et al. and Khan et al., i.e., increasing azithromycin MICs over the years for typhoidal salmonellae and non-agreement between disc diffusion and E-test susceptibility results for a significant (16.6%) number of isolates. Since the E-test method appears to be more reliable than disc diffusion, a switch to E-test may be warranted for azithromycin susceptibility of typhoidal salmonellae in endemic countries to avoid reporting false resistance.

Goldblatt et al. demonstrated that the use of E-strip for azithromycin MICs may be prone to reader bias errors [25,26]. This can lead to significant differences between local and reference laboratories resulting in over-reporting of resistance. To overcome this, prior training at the institute level and a “second reader system” have been proposed. We adopted this system to minimize reading errors in our study. The importance of this second reader system has also been highlighted by Skittrall et al. where clinical decision-making in two patients faced challenges due to false reporting of azithromycin resistance by the E-strip method [19]. Unfortunately despite being susceptible, repeatedly there have been reports of clinical failure with azithromycin [27].

There are several limitations to our study. First, it is a single-center study with a small sample size performed over a short period of time and may represent certain strains circulating in the local population. Second, we did not perform the molecular analysis of resistant isolates and those with high MICs. Third, the number of true resistant isolates was very small to exactly know the sensitivity of the disc diffusion method. Fourth, we did not monitor the clinical response of patients on azithromycin in our study. Nonetheless, we believe that our findings are important owing to few studies on azithromycin MICs. Further studies at a large scale with a large number of isolates are needed for the evaluation of disc diffusion breakpoints for azithromycin susceptibility and their correlation with E-test MICs. Although E-test MICs are expensive as compared to disc diffusion tests but considering the reliability of the E-test method it would be a cost-effective method in the longer run.

## Conclusions

Azithromycin resistance in isolates of *S. typhi* is increasing and it may present a threat to clinicians especially in typhoid endemic countries where XDR *S. typhi* is endemic now. Antimicrobial stewardship especially in the post-COVID-19 era is essential to prevent the development of resistance to this limited oral choice against XDR typhoid. Disc diffusion method may not be a reliable option for azithromycin susceptibility, especially, in XDR typhoid endemic areas. We recommend the routine use of E-strip MICs for azithromycin susceptibility at least in typhoid endemic areas.
